# Integrative transcriptomic and metabolomic analysis to elucidate the effect of gossypol on *Enterobacter* sp. GD5

**DOI:** 10.1371/journal.pone.0306597

**Published:** 2024-08-06

**Authors:** CaiDie Wang, XiaoBin Li, Jun Pan, Chen Ma, ShiQi Zhang, Changjiang Zang, KaiLun Yang

**Affiliations:** Xinjiang Key Laboratory of Herbivore Nutrition for Meat & Milk, College of Animal Science, Xinjiang Agricultural University, Urumqi, Xinjiang, China; ICAR - Central Institute for Research on Cotton Technology, INDIA

## Abstract

Gossypol, a yellow polyphenolic compound found in the *Gossypium* genus, is toxic to animals that ingest cotton-derived feed materials. However, ruminants display a notable tolerance to gossypol, attributed to the pivotal role of ruminal microorganisms in its degradation. The mechanisms of how rumen microorganisms degrade and tolerate gossypol remain unclear. Therefore, in this study, *Enterobacter* sp. GD5 was isolated from rumen fluid, and the effects of gossypol on its metabolism and gene expression were investigated using liquid chromatography-mass spectrometry (LC-MS) and RNA analyses. The LC-MS results revealed that gossypol significantly altered the metabolic profiles of 15 metabolites (eight upregulated and seven downregulated). The Kyoto Encyclopedia of Genes and Genomes analysis results showed that significantly different metabolites were associated with glutathione metabolism in both positive and negative ion modes, where gossypol significantly affected the biosynthesis of amino acids in the negative ion mode. Transcriptomic analysis indicated that gossypol significantly affected 132 genes (104 upregulated and 28 downregulated), with significant changes observed in the expression of catalase peroxidase, glutaredoxin-1, glutathione reductase, thioredoxin 2, thioredoxin reductase, and alkyl hydroperoxide reductase subunit F, which are related to antioxidative stress. Furthermore, Gene Ontology analysis revealed significant changes in homeostatic processes following gossypol supplementation. Overall, these results indicate that gossypol induces oxidative stress, resulting in the increased expression of antioxidative stress-related genes in *Enterobacter* sp. GD5, which may partially explain its tolerance to gossypol.

## Introduction

Cottonseed is recognized for its high energy [1], protein [2], and fiber [3], making it an advantageous feed component for ruminants [4] and a promising ingredient for fish diets [5]. However, cottonseed, cottonseed hull, and other cotton byproducts contain large amounts of the secondary metabolite gossypol, which hinders their direct application as feed materials. Numerous studies have reported that ruminants show no adverse symptoms after ingesting gossypol-containing cotton byproducts [6, 7]; however, gossypol can lead to liver injury and growth inhibition in ducks [8]. Suggesting that the different responses between ruminants and monogastric animals may be attributed to rumen-specific microorganisms. The rumen microbiome plays an important role in gossypol degradation, which occurs within 6 h of ingestion [9]. After anaerobic fermentation of cotton meal by *Bacillus* spp. isolated from rumen, the degradation rate of free gossypol was found to reach 93.46%, with an acid-soluble protein content of 13.26% [10]. The rumen-derived *Lact*. *mucosae* LLK-XR1 strain showed 40.65% gossypol degradation and good survivability at pH 3.0 and 0.3% bile [11]. Therefore, studying gossypol tolerance and degradation by rumen microorganisms may enable the future use of cotton byproducts for ruminants.

In a previous study, [14C] gossypol was orally administered to pigs, and metabolites isolated from their livers were characterized as gossypol, gossypolone, gossypolonic acid, demethylated gossic acid, and apogossypol [12]. The ruminal degradation mechanism of gossypol involves its binding to soluble proteins [13]. However, mechanisms underlying the selectivity of the rumen *Enterobacter* sp. GD5 strain for gossypol biodegradation and tolerance remains unclear. Therefore, in this study, we aimed to identify gossypol-degrading bacteria using gossypol as the sole carbon source and to elucidate the effects of gossypol on the metabolic and transcriptional activities of rumen *Enterobacter* sp. GD5. This study provides a preliminary exploration of the mechanisms underlying the tolerance of rumen microbiota to gossypol in ruminants.

## Materials and methods

### Strains

**Fig 1:**
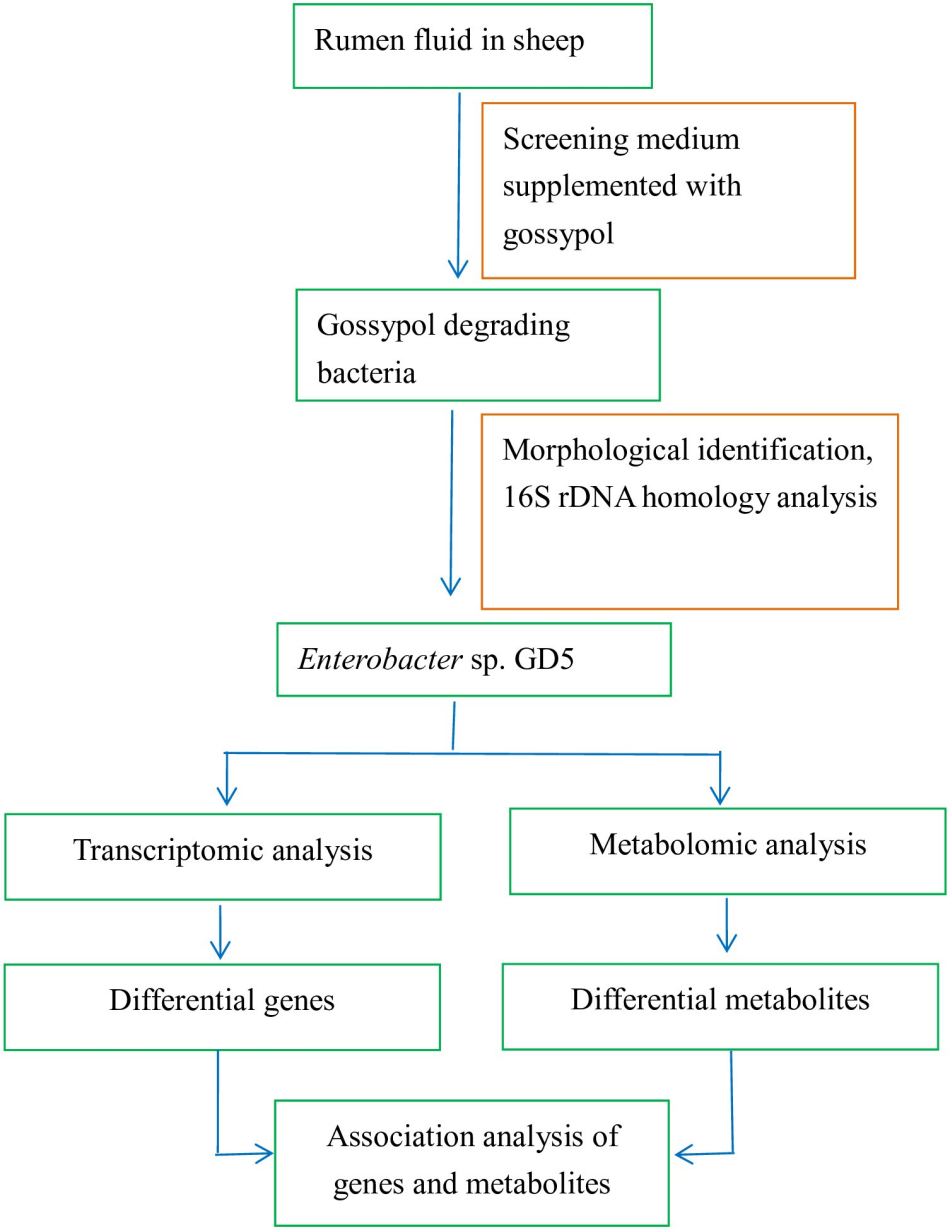
Flowchart of experimental.

The gossypol-degrading *Enterobacter* sp. GD5 strain was isolated from rumen fluid in sheep and screened through a medium supplemented with gossypol (Shanghai Kewei Chemical Technology Co., Ltd., Shanghai, China) as the sole carbon source (0.95 g/L (NH_4_)_2_SO_4_, 0.15 g/L gossypol, 1.36 g/L Na_2_HPO_4_, 1.48 g/L KHPO_4_, and 0.143 g/L MgSO_4_.7H_2_O). Thalli of the isolated strain were inoculated into liquid Luria-Bertani (LB) broth and cultured for 24 h at 39°C, and then harvested from the liquid medium via centrifugation. DNA was extracted using the sodium dodecyl sulfate-proteinase K method. The genomic DNA was amplified by PCR using the universal primers, 27F: 5′-AGAGTTTGATCCTGGCTCAG-3′ and 1492R: 5′-GGTTACCTTGTTACGACTT-3′. The 16S rRNA genes of the isolated strain were sequenced by Shanghai Shenggong (Shanghai, China). The sequences were compared with reported sequences in the National Center for Biotechnology Information (NCBI) database using the Basic Local Alignment Search Tool hosted on the NCBI website. A phylogenetic tree was constructed using the neighbor joining method. Phylogenetic and molecular evolutionary analyses were conducted with 1000 bootstrap values using MEGA software (version 5.0; Biodesign Institute, Arizona State University, Tempe, AZ, USA). The strain was stored at −80°C in LB medium with 25% glycerol (v/v) at the Xinjiang Key Laboratory of Herbivore Nutrition for Meat & Milk (College of Animal Science, Xinjiang Agricultural University, Urumqi, China) (Fig 1).

### Media preparation and growth conditions

LB medium was used to culture *Enterobacter* sp. GD5. The medium was heated to 39°C and passed through CO_2_ until it became colorless (1 mL 0.1% resazurin was added), after which 78 mL LB medium was added into 100 mL anaerobic bottles. The LB medium was sterilized at 121°C for 20 min, then 0.298 g/L NaS (reducer) was added before use to maintain anaerobic conditions. Cultivations were performed at 39°C in a constant temperature incubator (Spring Instrument. Co. Ltd., Jiangsu, China).

### Preparation of metabolome and transcriptome samples

Three groups were created for metabolome and transcriptome analyses. In the gossypol group (Gossypol), 1 mL *Enterobacter* sp. GD5 (1 × 10^7^ CFU/mL; cultured overnight) and 1 mL gossypol (dissolved in acetone to a final concentration of 150 μg/mL) were transferred to 78 mL LB medium and cultured. In the CK group, 1 mL LB medium (without *Enterobacter* sp. GD5) and 1 mL gossypol (dissolved in acetone to a final concentration of 150 g/mL) were transferred to 78 mL LB and cultured. In the control group (Control), 1 mL *Enterobacter* sp. GD5 (1 × 10^7^ CFU/mL; cultured overnight) and 1 mL acetone were transferred to 78 mL LB medium and cultured. Samples for the Gossypol and CK groups were collected at 0, 24, 48, 72, 96, 120, and 144 h, and the gossypol concentration in the medium was determined using high-performance liquid chromatography (HPLC; Shimadzu, Kyoto, Japan) [14]. Bacteria in the Gossypol and Control groups were incubated at 37°C for 72 h under anaerobic conditions and then centrifuged at 12,000 × *g* for 10 min at 4°C. The supernatant was collected and the sediments of *Enterobacter* sp. GD5 were analyzed using LC-MS. The experiments were repeated three times, with six and seven biological samples used for transcriptomic and metabolomic analyses, respectively.

### Metabolomics analysis

A 100 μL sample of each supernatant was extracted with 500 μL of 80% methanol aqueous solution containing 0.1% formic acid in a centrifuge tube and then vortexed for 30 s, followed by a 5 min ice bath. The samples underwent centrifugation at 15,000 × g for 10 min at 4 °C. The methanol content of the samples was diluted to 60% with MS-grade water. The filtrate was centrifuged in a centrifuge tube with a 0.22-μm filter at 15,000 × g and for 10 min at 4°C, followed by LC-MS analysis.

LC-MS analysis was performed using an ultra-HPLC system (Thermo Fisher Scientific, Waltham, MA, USA) with a Hypersil GOLD column (C18; 250 × 4.6 mm, 5 mm particle size) at 40°C coupled with a QE-HF-X system (Thermo Fisher Scientific, Waltham, MA, USA). The mobile phase (flow rate: 1 mL/min) comprised 0.1% formic acid and methanol in the positive mode and 5 mM ammonium acetate (pH 9.0) and methanol in the negative mode. The gradient elution procedure was as follows: 0 min, 98% A; 1.5 min, 98% A; 12 min, 0% A; 14 min, 0% A; 14.1 min, 98% A; and 16 min, 98% A. The MS scanning range was 100–1500 m/z, with the following electron spray ionization settings: spray voltage, 3.2 kV; sheath gas flow rate, 35 arb; auxiliary gas flow rate, 10 arb; capillary temperature, 320 °C; and polarity, positive and negative ion modes. The MS/MS secondary scans were data-dependent.

Principal component analysis (PCA) and partial least squares discriminant analysis (PLS-DA) were used to analyze the effects of gossypol on rumen metabolites (Gossypol vs. Control groups). The Variable Importance in the Projection (VIP) value of the first principal component (PC1) of the PLS-DA model was used to identify differential metabolites by combining the *P*-values of the t test. The threshold was set to VIP > 1.0, a fold change (FC) >1.2 or FC < 0.833, and *P* < 0.05.

### RNA isolation, quality control, and library construction

Total RNA from *Enterobacter* sp. GD5 was extracted with liquid nitrogen and added to TRIzol solution. An Agilent 2100 biological analyzer (Agilent Technologies, Santa Clara, CA, USA) was used to detect RNA quality and concentration. The NovaSeq 6000 platform (Illumina, San Diego, CA, USA) was used for sequencing obtained raw data. Raw transcriptomic data were deposited in the NCBI Sequence Read Archive (BioProject accession number: PRJNA616020).

Clean reads were obtained by removing those containing adapter or ploy-N sequences, and low-quality reads to ensure the downstream analysis of high-quality data. The reference genome of the *Enterobacter hormaechei* WCHEH020038 (CP031726.1) was downloaded from the genome website. Clean reads were aligned to the reference genome using Bowtie software (version 2–2.2.3; http://bowtie-bio.sourceforge.net/index.shtml).

Differential expression analysis between the two groups was performed using the DESeq R package (version 1.18.0; https://bioconductor.org/packages/release/bioc/html/DESeq.html). *P*-values were adjusted using the Benjamini—Hochberg approach to control the false discovery rate. Gene Ontology (GO0 and Kyoto Encyclopedia of Genes and Genomes (KEGG) analyses of differentially expressed genes (DEGs) were performed using the “GOseq” package in R (https://bioconductor.org/packages/release/bioc/html/goseq.html), correcting for gene-length bias. GO terms with a corrected *P*-value < 0.05 were considered significantly enriched. KOBAS software (http://kobas.cbi.pku.edu.cn/kobas3/) was used to test the statistical enrichment of DEGs in the KEGG pathways.

### Reverse transcription-PCR analysis

The expression levels of six genes (*katG*, *dps*, *poxB*, *lsrA*, *UfaA1*, and *hemH*) were detected via reverse transcription PCR using an iQ5 system (Bio-Rad, Hercules, CA, USA). The primers used are listed in S1 Table. The PCR program was conducted as follows: 95°C for 1 min, followed by 30 cycles at 95°C for 10 s, 65°C for 20 s, and 72°C for 20 s. Expression levels were normalized against the 16S rRNA gene using the 2^−ΔΔCt^ method [15, 16].

## Results

### Isolation and identification of gossypol degrading bacteria

**Fig 2:**
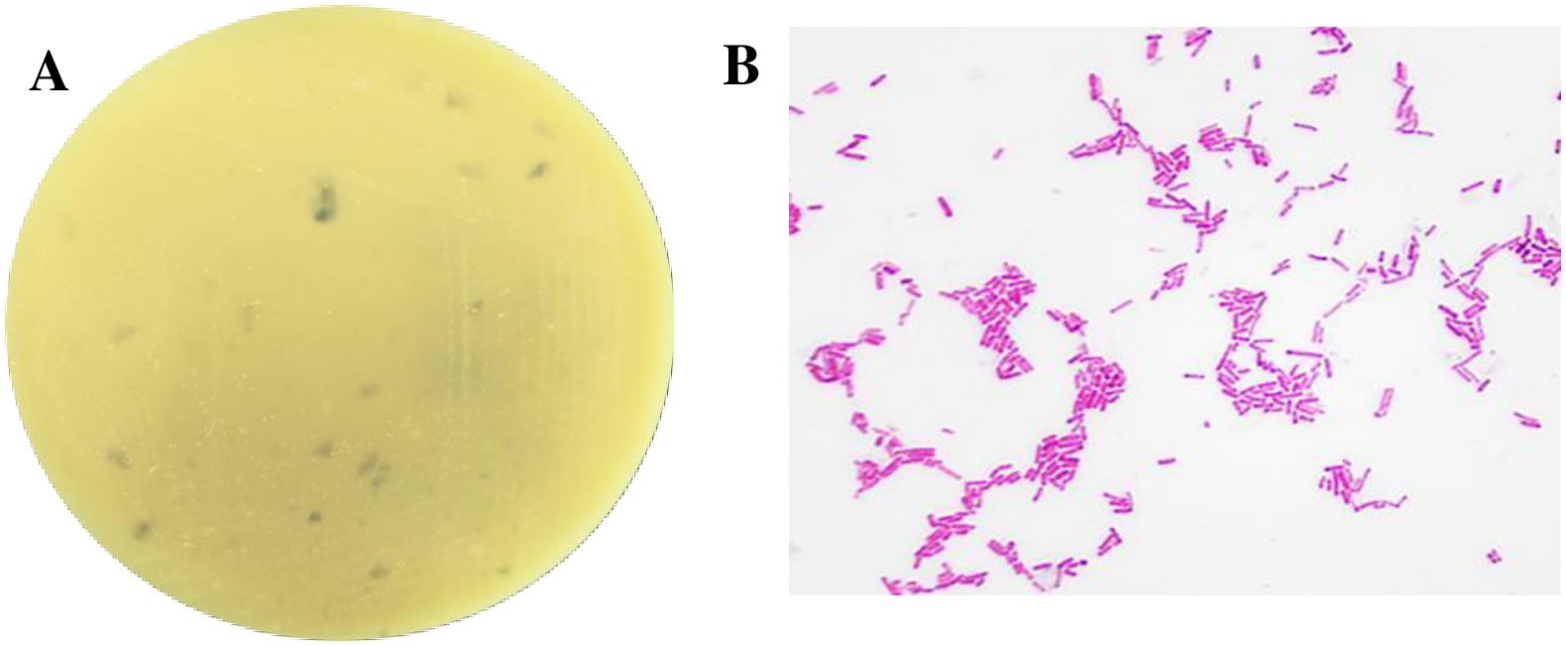
Morphological identification of the gossypol-degrading strain. (A) Colony and (B) microstructure characteristics of the strain (1000× magnification).

A single strain was isolated from the rumen fluid of sheep using basal medium plates containing gossypol as the sole carbon source after 3 d of anaerobic fermentation (Fig 2A). The emergent colonies exhibited a round morphology and a white coloration. Gram staining procedures subsequently verified these bacteria as gram-negative (Fig 2B).

### 16S rDNA homology analysis

**Fig 3:**
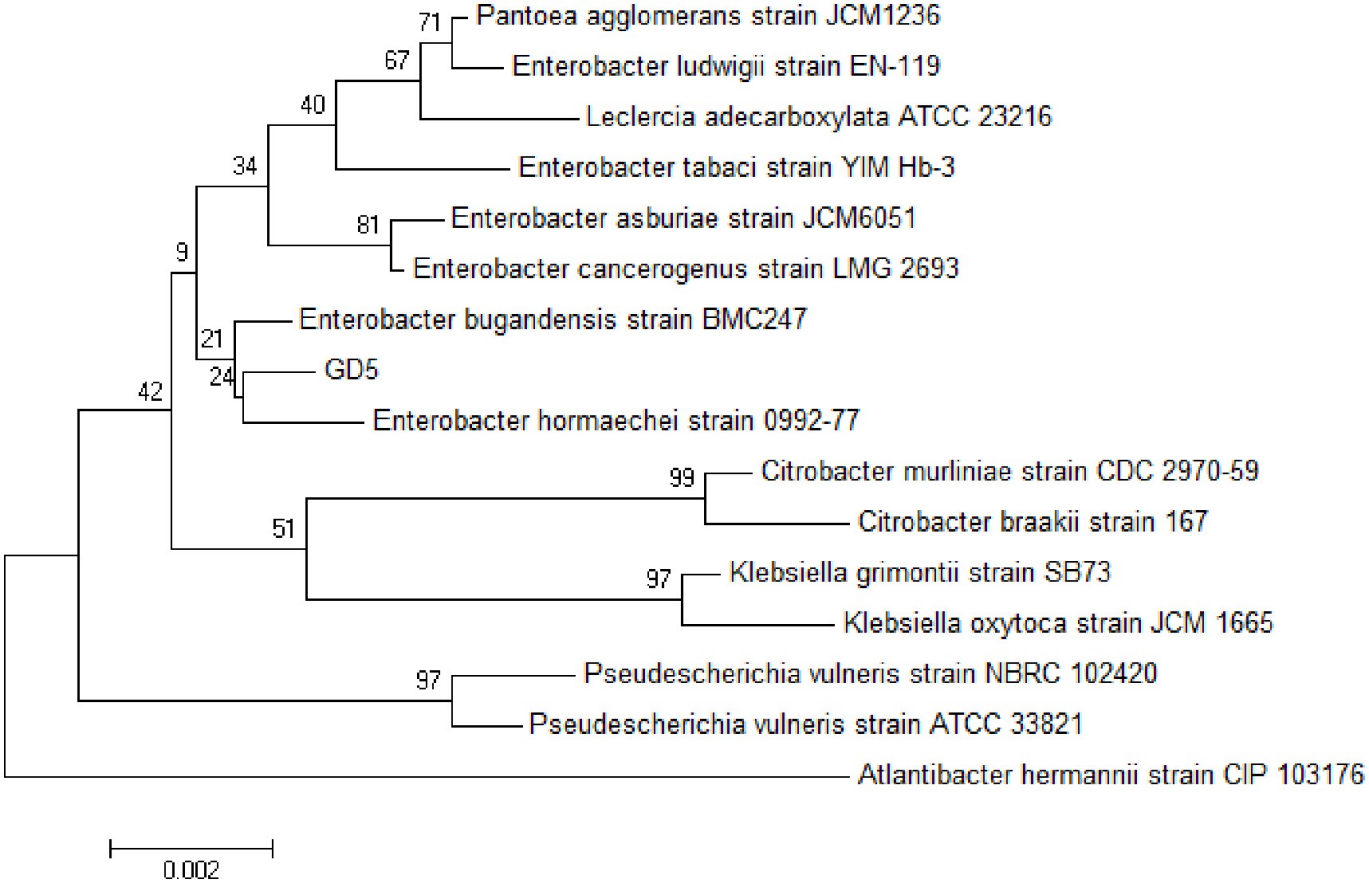
Phylogenetic tree of the 16S rDNA of the gossypol-degrading bacterium, GD5. Changes in gossypol concentration after fermentation.

The genomic DNA of the isolated strain was successfully extracted and amplified using a bacteria-specific primer pair, yielding an amplicon of approximately 1.5 kb, a method previously applied for various bacterial strains. Sequence analysis determined the amplicon size to be 1356 bp. Alignment of the 16S rRNA gene sequences of the isolated strain against the NCBI BLAST database demonstrated a 99% homology with the *Enterobacter hormaechei* 0992–77 strain (Fig 3).

**Fig 4:**
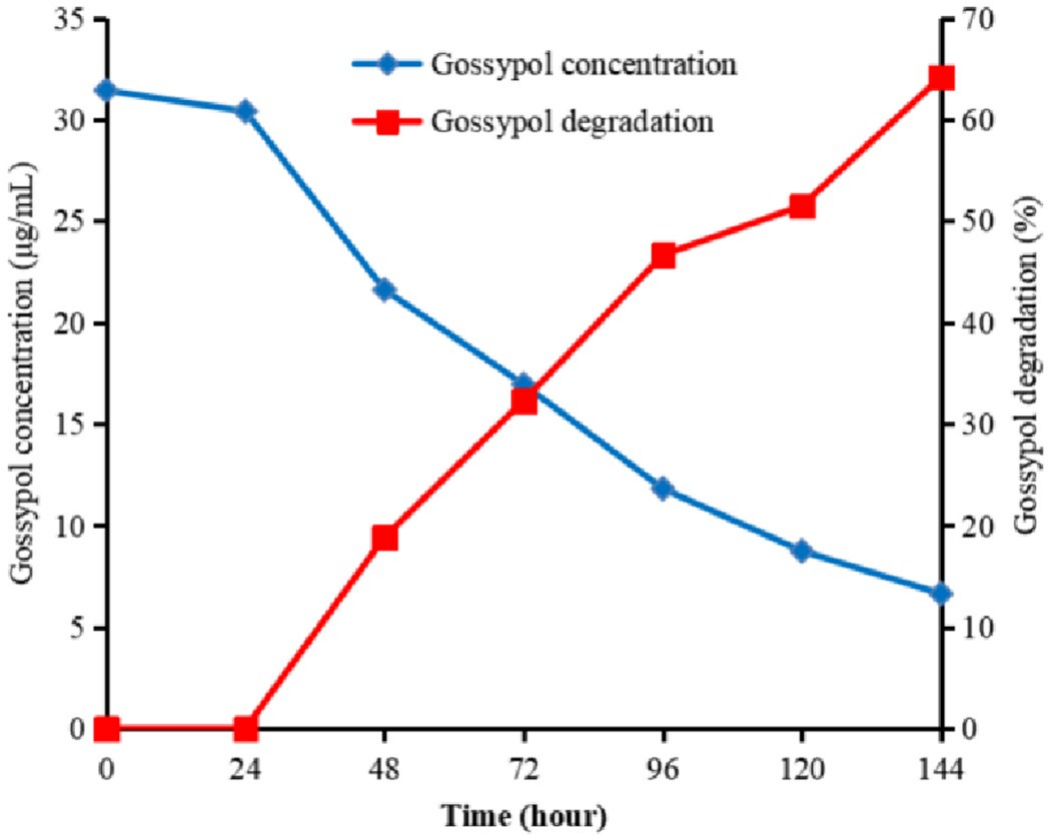
Concentration of gossypol in culture medium and its degradation by *Enterobacter* sp. GD5 at different times.

The concentration of gossypol in the culture medium was measured using HPLC. Results indicated the presence of 31.44 μg/mL gossypol before fermentation and 16.95 μg/mL after a 72-h culture, translating to a gossypol degradation rate of 32.31% (Fig 4). After 120 h, the degradation rate decreased.

### Metabolomic analysis of *Enterobacter* sp. GD5

PCA results of the positive and negative ion modes showed a distinct separation between the Gossypol and Control groups (S1A and S1D Fig). The PLS-DA results revealed a clear separation between the Gossypol and Control groups (S1B and S1E Fig), with R^2^ and Q^2^ values of 0.99 and 0.89, and 0.96 and 0.59, respectively. The permutation test R^2^ and Q^2^ values were 0.76 and −0.83 in the positive ion mode and 0.75 and −0.80 in the negative ion mode (S1C and S1F Fig), respectively.

The VIP of the PC1 of the PLS-DA model was used to identify differentially expressed metabolites according to a VIP threshold > 1.0, FC >1.2 or < 0.833, and *P* < 0.05. In the positive ion mode, 129 metabolites were detected (S2 Table), nine of which were significantly different (four metabolites were upregulated and five were downregulated). In the negative ion mode, 117 metabolites were detected (S3 Table), six of which were significantly different (four upregulated and two downregulated). Differential metabolites identified in the positive and negative ion modes are listed in Table 1.

**Table 1:** Differential metabolites identified in the culture medium of *Enterobacter* sp. GD5.

ID	Metabolites	Formula	Molecular Weight	RT (min)	FC
**Positive ion mode**					
Com_2733_pos	2-(14,15-epoxyeicosatrienoyl) glycerol	C_23_H_38_O_5_	376.259	14.843	6.852
Com_528_pos	Cadaverine	C_5_H_14_N_2_	102.116	1.099	1.943
Com_6022_pos	Cumene hydroperoxide	C_9_H_12_O_2_	152.084	13.233	1.898
Com_892_pos	L-(+)-citrulline	C_6_H_13_N_3_O_3_	175.096	1.292	1.439
Com_5587_pos	Alanyl-N-(6-amino-2-pyridinyl)-alpha-glutamine	C_13_H_19_N_5_O_4_	309.143	1.374	−0.771
Com_1049_pos	L-5-hydroxytryptophan	C_11_H_12_N_2_O_3_	220.085	2.987	−0.765
Com_2315_pos	Norharman	C_11_H_8_N_2_	168.069	7.959	−0.765
Com_13280_pos	5’-Deoxy-5’-((3-(methylammonio)propyl)amino)adenosine	C_14_H_25_N_7_O_3_	339.203	8.686	−0.682
Com_3252_pos	Zeatin-7-N-glucoside	C_16_H_23_N_5_O_6_	381.170	8.541	−0.621
**Negative ion mode**					
Com_201_neg	7Z-heptadecenoic acid	C_17_H_32_O_2_	268.240	14.6	6.365
Com_276_neg	2-oxobutyric acid	C_4_H_6_O_3_	102.032	1.368	2.151
Com_705_neg	L-ornithine	C_5_H_12_N_2_O_2_	132.090	1.306	1.495
Com_868_neg	Citrulline	C_6_H_13_N_3_O_3_	175.095	1.31	1.424
Com_167_neg	2-hydroxycaproic acid	C_6_H_12_O_3_	132.078	3.481	−0.739
Com_43_neg	Glyceraldehyde	C_3_H_6_O_3_	90.032	1.278	−0.649

FC = fold change; RT = reaction time.

KEGG enrichment analysis identified significantly different metabolites in the positive ion mode that participated in glutathione metabolism, lysine degradation, and protein digestion (Table 2). Metabolites identified in negative ion mode were enriched in 10 KEGG pathways.

**Table 2:** Kyoto Encyclopedia of Genes and Genomes (KEGG) enrichment of differential metabolites identified in *Enterobacter* sp. GD5.

Map ID	Pathway	*P*-value	Adjusted *P*-value
**Positive ion mode**			
map00480	Glutathione metabolism	0.077	0.205
map00310	Lysine degradation	0.103	0.205
map04974	Protein digestion, and absorption	0.205	0.274
**Negative ion mode**			
map01230	Biosynthesis of amino acids	0.004	0.047
map00220	Arginine biosynthesis	0.018	0.106
map01210	2-oxocarboxylic acid metabolism	0.059	0.133
map00290	Valine, leucine, and isoleucine biosynthesis	0.067	0.133
map00472	D-arginine and D-ornithine metabolism	0.067	0.133
map00480	Glutathione metabolism	0.067	0.133
map00260	Glycine, serine, and threonine metabolism	0.130	0.195
map00270	Cysteine and methionine metabolism	0.130	0.195
map00640	Propanoate metabolism	0.191	0.229
map00330	Arginine and proline metabolism	0.356	0.388

### Analysis of the *Enterobacter* sp. GD5 transcriptome

Total RNA isolated from *Enterobacter* sp. GD5 resulted in 23.98 million clean reads from 12 samples. The results revealed that 82.19–96.23% of the clean reads matched the reference genome (S2 Fig), suggesting that the transcriptome data are useful for further analysis. There were 132 DEGs identified between the Gossypol and Control groups, of which 104 were upregulated (S4 Table) and 28 downregulated (S5 Table). Swiss-Prot annotation identified genes related to homeostatic processes, antioxidative stress, and dehydrogenase activity (Table 3). Seven DEGs were involved in homeostatic processes, most of which were associated with antioxidative stress, and all DEGs were significantly upregulated.

**Table 3:** Differential gene expression in *Enterobacter* sp. GD5 (Gossypol vs. Control groups).

Gene ID	log_2_ FC	Gene description
Homeostatic processes		
D0Z05_15120	1.589	Glutathione reductase (*gorA*)
D0Z05_23255	2.264	DNA protection during starvation protein (*dps*)
D0Z05_23495	2.989	Glutaredoxin-1 (*grxA*)
D0Z05_10560	2.775	Thioredoxin-2 (*trxC*)
D0Z05_22325	2.946	Alkyl hydroperoxide reductase subunit F (*ahpCF*)
D0Z05_05310	2.686	Thiol:disulfide interchange protein (*dsbG*)
D0Z05_19845	1.195	Na(+)/H(+) antiporter (*NhaA*)
Antioxidative stress		
D0Z05_15675	0.279	Hydrogen peroxide-inducible genes activator(*oxyR*)
D0Z05_23495	2.989	Glutaredoxin-1 (*grxA*)
D0Z05_22325	2.946	Alkyl hydroperoxide reductase subunit F (*ahpCF*)
D0Z05_15715	3.536	Catalase peroxidase (*katG*)
D0Z05_15120	1.589	Glutathione reductase (*gorA*)
D0Z05_23790	1.885	Thioredoxin reductase (*trxB*)
D0Z05_10560	2.775	Thioredoxin 2 (*trxC*)
Dehydrogenase		
D0Z05_13800	−1.170	Glutarate-semialdehyde dehydrogenase (*davD*)
D0Z05_17860	−1.311	Isocitrate dehydrogenase kinase/phosphatase (*aceK*)
D0Z05_23705	−1.364	Pyruvate dehydrogenase (*poxB*)
D0Z05_04665	−1.477	3-hydroxyadipyl-CoA dehydrogenase(*paaH*)
D0Z05_15530	−1.520	Putative L-lactate dehydrogenase operon regulatory protein(*lldR*)
D0Z05_18180	−1.638	Formate dehydrogenase H (*fdhF*)
D0Z05_03355	−1.655	N-succinylglutamate 5-semialdehyde dehydrogenase (*astD*)
D0Z05_11900	−1.780	2-dehydro-3-deoxy-D-gluconate 5-dehydrogenase (*kduD*)
D0Z05_10780	−2.567	Histidinol dehydrogenase 2 (*hisD2*)
D0Z05_12280	−2.906	Phenylacetaldehyde dehydrogenase (*styD*)
D0Z05_12285	−3.028	Probable L-aspartate dehydrogenase (*nadX*)
D0Z05_12325	−3.542	Pyridoxal 4-dehydrogenase (*pldh-t*)

**Fig 5:**
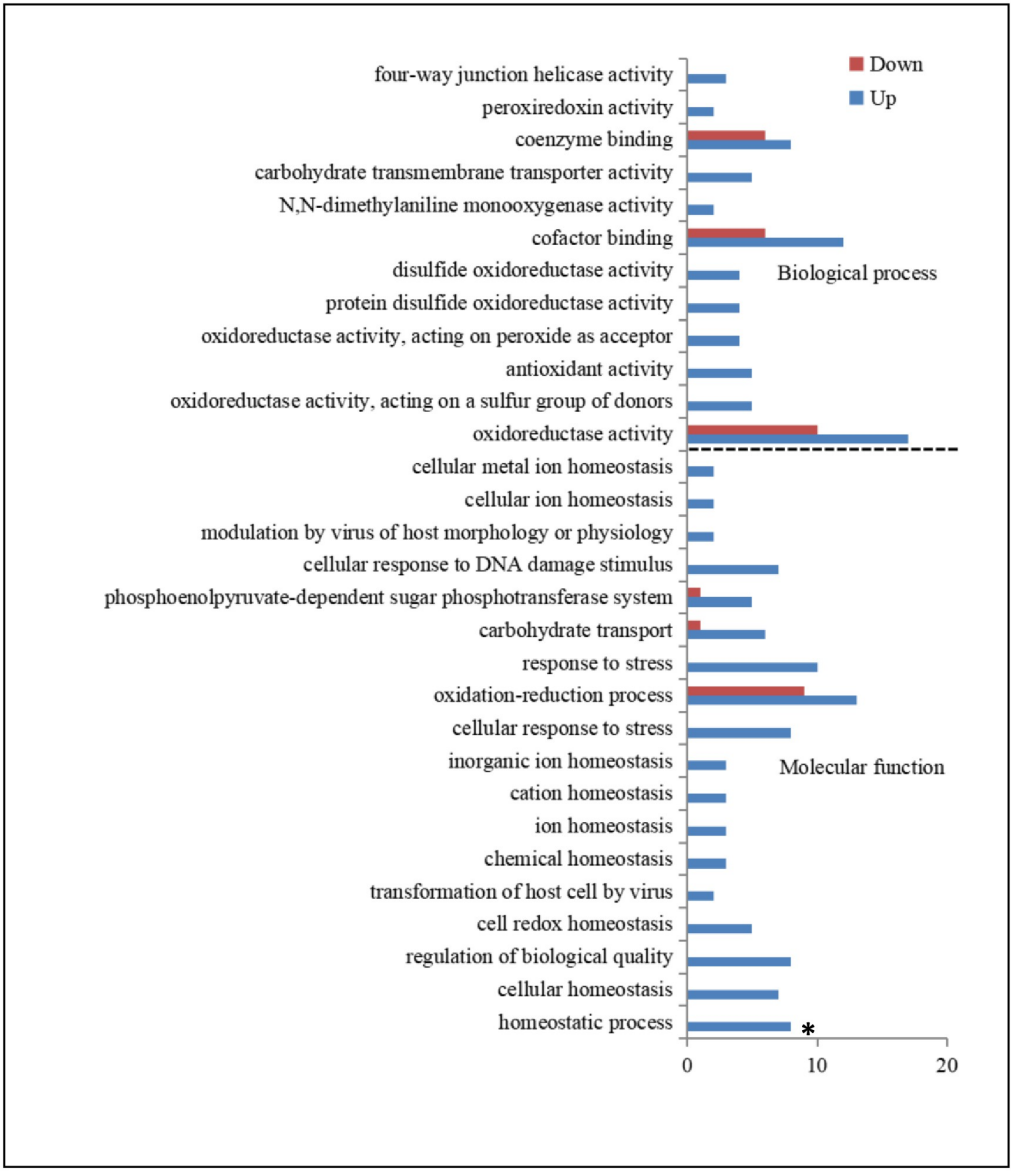
Gene Ontology (GO) enrichment of DEGs following gossypol treatment. The top 30 GO terms are shown. *P <0.05.

The GO enrichment analysis is results are shown in Fig 5. The 30 GO terms with the most significant enrichment were selected. DEGs were mainly enriched in biological processes and molecular functions, with the most highly enriched genes involved in oxidoreductase and oxidation-reduction activities. Genes involved in homeostatic processes differed significantly between the Gossypol and Control groups.

KEGG enrichment analysis showed that the DEGs were associated with various cellular processes (Table 4). The RT-qPCR was used to validate the gene expression profiles. Results showed that the expression trends of the selected genes were the same as those detected via RNA-seq (S3 Fig).

**Table 4:** KEGG pathway enrichment of DEGs.

ID	Pathway	*P*-value	Adjusted *P*-value
eclg00620	Pyruvate metabolism	0.012	0.218
eclg00450	Selenocompound metabolism	0.013	0.218
eclg00030	Pentose phosphate pathway	0.016	0.218
eclg00380	Tryptophan metabolism	0.037	0.228
eclg00010	Glycolysis/gluconeogenesis	0.039	0.228
eclg00640	Propanoate metabolism	0.041	0.228
eclg00520	Amino sugar and nucleotide sugar metabolism	0.045	0.228
eclg02060	Phosphotransferase system	0.045	0.228

### Association analysis between the metabolome and transcriptome

**Fig 6:**
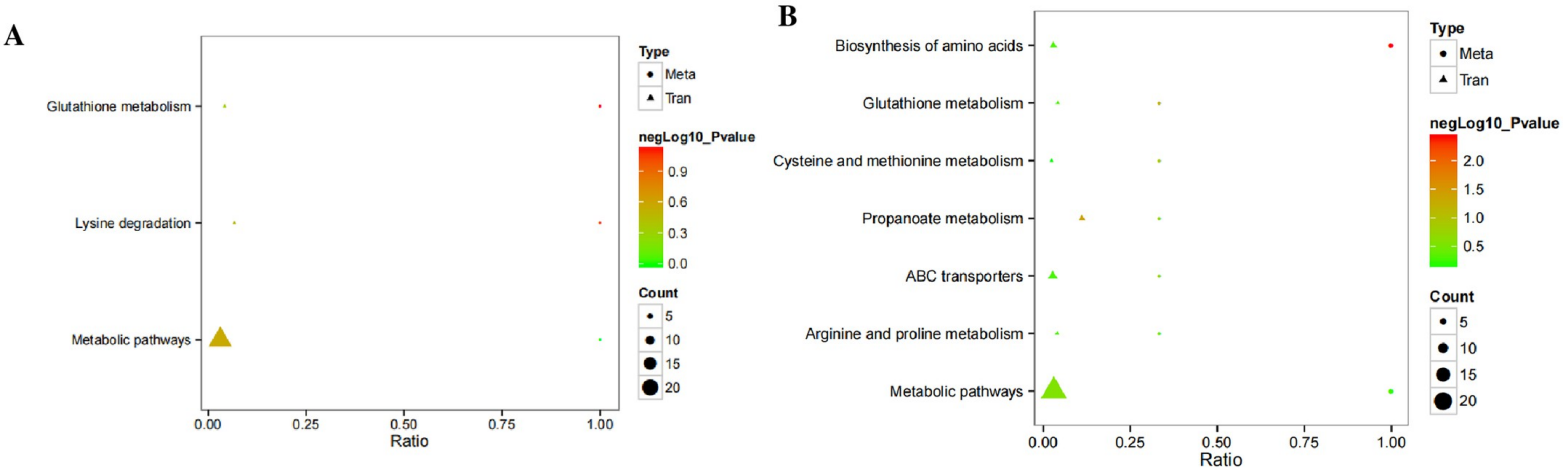
Integrative metabolomic and transcriptomic association analysis in *Enterobacter* sp. GD5. (A) Positive ion mode; (B) negative ion mode. "Meta" denotes metabolism, with circles illustrating metabolic data; "Tran" refers to transcription, with triangles indicating gene data. "P-value" indicates the statistical significance within transcriptional or metabolic pathways, while "Count" indicates the quantity of metabolites or genes involved.

Analysis of the association between the metabolome and transcriptome sequences showed that significantly different metabolites and genes participated in glutathione metabolism, lysine degradation, and other metabolic pathways in the positive ion mode (Fig 6A). However, in the negative ion mode, pathways were enriched in the biosynthesis of amino acids, glutathione metabolism, cysteine and methionine metabolism, propanoate metabolism, ABC transporters, arginine and proline metabolism, and other metabolic pathways (Fig 6B).

## Discussion

Previous studies have reported the important role of the rumen microbiome in gossypol degradation, which occurs within 6 h of ingestion [9]. Zhang et al. isolated a strain of *B*. *subtilis* from bovine rumen fluid and found that it degraded free and total gossypol by 78.86% and 49.00%, respectively [17]. In previous studies, a *Lactobacillus agilis* WWK129 strain was anaerobically isolated from dairy cows and exhibited gossypol degradability as high as 83% [18]. *Enterobacter hormaechei* ASU-01 strain degraded most of the naphthalene in culture medium within 15 days [19]. In the present study, the gossypol degrading bacterium *Enterobacter* sp. GD5 was isolated using a culture medium containing gossypol as the sole carbon source. The gossypol-degradation rate of *Enterobacter* sp. GD5 was measured as 32.31% after 72 h, which is much lower than that reported previously. This may be due to the different strains and culture conditions used in this study. Additionally, rumen microorganisms comprise a complex biological system and gossypol degradation may be caused by the synergistic activity of multiple microorganisms.

Numerous studies employed metabolomics to detect microbial metabolic changes [20, 21]. Gossypol exhibits reducing activity on lysine and other amino acids at rates of 0.003–1.03% [22]. The mechanism of gossypol degradation in the rumen involves the binding of each mole of gossypol to two moles of lysine ε-amino groups [13]. In the present study, amino acid biosynthesis was identified as a significantly differentially expressed metabolic pathway in gossypol-exposed *Enterobacter* sp. GD5. Additionally, KEGG analysis indicated that the glutathione pathway was enriched, according to results obtained in both the positive and negative ion modes. In prokaryotic cells, glutathione is a low-molecular-weight peptide (l-γ-glutamyl-l-cysteinylglycine) that protects cells as an antioxidant [23] and plays a crucial role in cellular defense against xenobiotics and naturally occurring deleterious compounds [24]. This suggests that glutathione may be involved in gossypol resistance and degradation in *Enterobacter* sp. GD5. A previous study identified various gossypol metabolites in the pig liver [25]; however, we did not identify any of those reported metabolites in the current study. This difference may be explained by the fact that the degradation of gossypol by *Enterobacter* sp. GD5 is different from that in the liver, and it is possible that the LC-MS analysis could not detect all metabolites.

**Fig 7:**
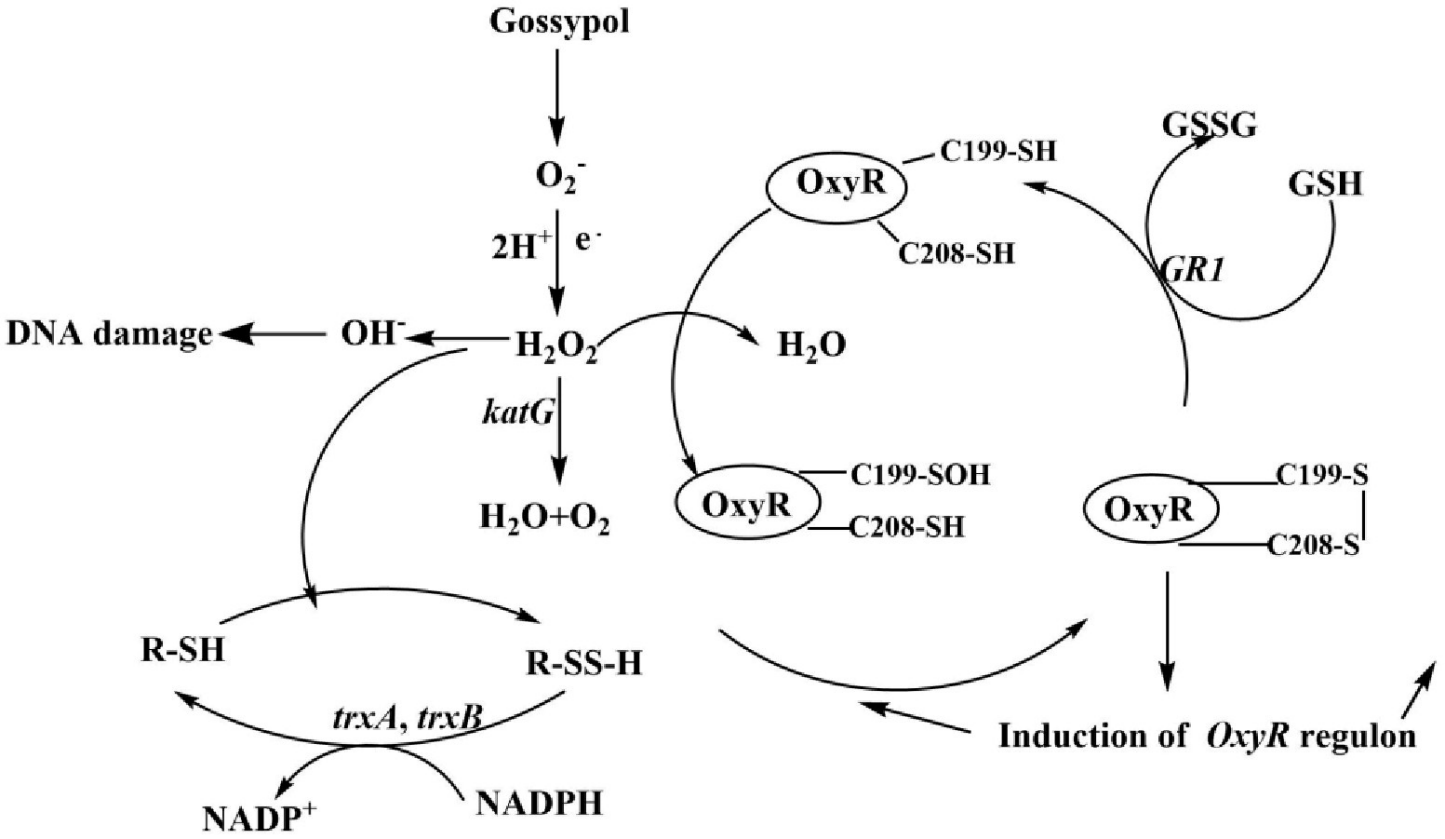
Mechanisms of gossypol-induced antioxidative stress in *Enterobacter sp*. GD5. Gossypol treatment differentially upregulated the expression of *katG*, *ahpCF*, *goxA*, *grxA* (GR1), *trxA*, and *trxB*.

Transcriptome analysis was performed to determine the influence of gossypol on gene expression in *Enterobacter* sp. GD5. Previous studies have used transcriptomic analysis to study changes in gene expression in *B*. *pumilus* during various growth phases [26]. Genomic, transcriptomic, and proteomic analyses have been used to reveal the possible mechanisms of environmental adaptation in *E*. *coli* [27]. Therefore, transcriptomics plays an important role in understanding microbial tolerance (or biodegradation). Gossypol as a promising antimicrobial compound that inhibits cell division by affecting the GTPase activity of filamenting temperature sensitive mutant Z (FtsZ) [28]. Anthraquinone-2,6-disulfonate promotes the expression of genes related to glutathione metabolism, reduces oxidative stress, and enhances *Enterobacter* sp. DNB-S2-mediated biodegradation of dibutyl phthalate [29]. In the present study, we found that gossypol influenced the homeostatic processes in *Enterobacter* sp. GD5. Most of the genes involved were related to antioxidative stress. Genes responding to oxidative stress included *katG*, *ahpCF*, *goxA*, *grxA* (GR1), and *dps* [30]. The *katG* gene *KatG* catalyzes the conversion of H_2_O_2_ to H_2_O and O_2_ and acts as a vanguard during the anti-peroxide process in *E*. *coli* [31]. Catalase HPI encoded by *KatG* protects the strain from high H_2_O_2_ concentrations. The *ahpCF* gene encodes a reductase that acts as an effective scavenger of low concentrations of H_2_O_2_ in *E*. *coli* [32]. GR1 catalyzes the reduction of intramolecular disulfide bonds (Fig 7). The two thioredoxins, thioredoxin 1 (Trx 1) and thioredoxin 2 (Trx 2), are typically present in *E*. *coli* and are encoded by *trxA* and *trxC*, respectively [33]. Trx 1 and Trx 2 are involved in reducing disulfide bonds [34] and can degrade 2,4-dichlorophenoxyacetic acid, a toxic herbicide [35]. Microbial antioxidant systems have been extensively studied, and major regulons, such as *oxyR*, *soxR*, *rpoS*, *perR*, *ohrR*, and *σв*, significantly contribute to enhancing the oxidative tolerance of microorganisms. A series of antioxidant genes, including *dps*, *katE*, *xthA*, *sodC*, ahpC*/F*, *mrgA*, and *fur*, are under the control of these regulators [36]. Overall, these results revealed that gossypol likely induces oxidative stress in *Enterobacter* sp. GD5, where the expression of genes related to antioxidative stress was significantly increased.

Gossypol affects enzymatic activity by forming Schiff bases. A previous study reported that gossypol inhibits pepsinogen at a concentration of 4.7 × 10^−5^ mol/L [37]. Gossypol also attenuates the growth of *Trypanosoma cruzi* by inhibiting α-hydroxy-acid dehydrogenase and malate dehydrogenase activities [38]. Catalase and glutathione reductase activities increased 1.5–2.0 fold in fungal mycelia grown in the presence of gossypol as a carbon source [39]. Santana et al. reported an increase in glutathione reductase activity in the testicular homogenate of rats that were fed gossypol [40]. Catalase and glutathione reductase levels are upregulated in *Aspergillus* in response to oxidative stress caused by toxicants, such as arsenic and lead, respectively [41]. Formate dehydrogenase, processes associated with iron ions, repair programs, multidrug resistance, antioxidant defense, and energy generation (*mqo* and *sdhC*) may have contributed to stress tolerance in the *Enterobacter* NRS-1 strain [42]. These findings are consistent with our results, which revealed the gossypol-mediated the downregulation of genes encoding formate dehydrogenase (*fdhF*). KEGG analysis identified pyruvate metabolism as the primary highly enriched pathway. The pyruvate metabolism-associated genes are mostly involved in the tricarboxylic acid cycle, cell growth rate, and substrate-to-biomass conversion efficiency [43]. Overall, these results suggest that gossypol alters nutrient metabolism and inhibits bacterial growth.

## Conclusions

Our metabolic results revealed that gossypol impacts glutathione metabolism in *Enterobacter* sp. GD5. The RNA-seq results revealed significant modifications to homeostatic processes within *Enterobacter* sp. GD5, with a considerable differential expression of genes associated with the antioxidative stress response. These findings contribute to our understanding of the mechanisms underlying the observed gossypol tolerance and degradation capabilities of *Enterobacter* sp. GD5. Future research should aim to identify additional gossypol-degrading microorganisms from the rumen, encompassing both bacterial and fungal species. Comparative studies on gossypol degradation or tolerance mechanisms across different microorganisms will enhance our knowledge of rumen microbial interactions with gossypol.

## Supporting information

S1 Table The primers of reverse transcription PCR. (DOCX)

S2 Table metabolites were detected in the positive ion mode. (XLS)

S3 Table metabolites were detected in the negative ion mode. (XLS)

S4 Table Differentially expressed genes (upregulated). (XLS)

S5 Table Differentially expressed genes (downregulated). (XLS)

S1 Fig PCA scatter plots, PLS-DA, and permutation tests between gossypol-treated and control groups. (DOCX)

S2 Fig Test results of total RNA. (DOCX)

S3 Fig Detection of relative gene expression by qRT-PCR. (DOC)

## References

[1] Chetima A, Wahabou A, Zomegni G, Ntieche Rahman A, Bup Nde D. Bleaching of neutral cotton seed oil using organic activated carbon in a batch system: Kinetics and adsorption isotherms. Processes (Basel). 2018;6:22. doi: 10.3390/pr6030022

[2] Ma M, Ren Y, Xie W, Zhou D, Tang S, Kuang M, et al. Physicochemical and functional properties of protein isolate obtained from cottonseed meal. Food Chem. 2018;240:856–862. doi: 10.1016/j.foodchem.2017.08.030 28946352

[3] Palmquist DL. Digestibility of cotton lint fiber and whole oilseeds by ruminal microorganisms. Anim Feed Sci Technol. 1995;56:231–242. doi: 10.1016/0377-8401(95)00830-6

[4] Underwood AG, Martin S, Greene W, Smith WB. Case study: Passage dynamics of whole cottonseed in beef cattle. J Anim Sci. 2023;101(Suppl 1):87. doi: 10.1093/jas/skad068.103

[5] Huang Y-J, Zhang N-N, Fan W-J, Cui Y-Y, Limbu SM, Qiao F, et al. Soybean and cottonseed meals are good candidates for fishmeal replacement in the diet of juvenile *Macrobrachium nipponense*. Aquac Int. 2018;26:309–324. doi: 10.1007/s10499-017-0215-1

[6] Câmara ACL, do Vale AM, Mattoso CRS, Melo MM, Soto-Blanco B. Effects of gossypol from cottonseed cake on the blood profile in sheep. Trop Anim Health Prod. 2016;48:1037–1042. doi: 10.1007/s11250-016-1039-0 27098313

[7] Leite RG, Hoffmann A, Romanzini EP, Delevatti LM, Ferrari AC, Fonseca NVB, et al. Zebu cattle fed dry distiller’s grain or cottonseed meal had greater nitrogen utilization efficiency than non-supplemented animals. Trop Anim Health Prod. 2022;54(2):119. doi: 10.1007/s11250-022-03126-6 35226193

[8] Yildirim M, Lim C, Wan PJ, Klesius PH. Growth performance and immune response of channel catfish (*Ictalurus puctatus*) fed diets containing graded levels of gossypol—acetic acid. Aquaculture. 2003;219:751–768. doi: 10.1016/s0044-8486(03)00062-0

[9] Tang C-H, Liu J, Zhao Q-Y, Zhang J-M. Pharmacokinetic comparison of gossypol isomers in cattle: transfer from diet to plasma and degradation by rumen microbes. J Zhejiang Univ Sci B. 2018;19:471–480. doi: 10.1631/jzus.b1700289

[10] Li J, Gao T, Hao Z, Guo X, Zhu B. Anaerobic solid-state fermentation with *Bacillus subtilis* for digesting free gossypol and improving nutritional quality in cottonseed meal. Front Nutr. 2022;9. doi: 10.3389/fnut.2022.1017637 36570163 PMC9773203

[11] Lv L, Xiong F, Liu Y, Pei S, He S, Li S, et al. The rumen-derived *Lact*. *mucosae* LLK-XR1 exhibited greater free gossypol degradation capacity during solid-state fermentation of cottonseed meal and probiotic potential. BMC Microbiol. 2024;24. doi: 10.1186/s12866-023-03156-6 38183000 PMC10768434

[12] Abou-Donia MB, Dieckert JW. Metabolic fate of gossypol: The metabolism of [14C] gossypol in swine. Toxicol Appl Pharmacol. 1975;31:32–46. doi: 10.1016/0041-008x(75)90049-6 1129787

[13] Reiser R, Fu HC. The mechanism of gossypol detoxification by ruminant animals. J Nutr. 1962;76:215–218. doi: 10.1093/jn/76.2.215 14491326

[14] Cheng C, Cun-Xi N, Jing L, Yong-Qiang W, Yan-Feng L, Wen-Xia G, et al. Validated method to determine (±)-gossypol in *Candida tropicalis* culture by high-performance liquid chromatography. Acta Chromatogr. 2018;30:269–273. doi: 10.1556/1326.2018.00420

[15] Ji K, Wang W, Zeng B, Chen S, Zhao Q, Chen Y, et al. Bacterial cellulose synthesis mechanism of facultative anaerobe *Enterobacter* sp. FY-07. Sci Rep. 2016;6:21863. doi: 10.1038/srep21863 26911736 PMC4766428

[16] Guérin F, Isnard C, Cattoir V, Giard JC. Complex regulation pathways of AmpC-mediated β-lactam resistance in *Enterobacter cloacae* complex. Antimicrob Agents Chemother. 2015;59:7753–7761. doi: 10.1128/aac.01729-15 26438498 PMC4649247

[17] Zhang Y, Zhang Z, Dai L, Liu Y, Cheng M, Chen L. Isolation and characterization of a novel gossypol-degrading bacteria *Bacillus subtilis* strain Rumen *Bacillus subtilis*. Asian-Australas J Anim Sci. 2018;31:63–70. doi: 10.5713/ajas.17.0018 28728360 PMC5756925

[18] Wang W-K, Li W-J, Wu Q-C, Wang Y-L, Li S-L, Yang H-J. Isolation and identification of a Rumen Lactobacillus bacteria and its degradation potential of gossypol in cottonseed meal during solid-state fermentation. Microorganisms. 2021;9:2200. doi: 10.3390/microorganisms9112200 34835326 PMC8622920

[19] Hesham AE-L, Mawad AMM, Mostafa YM, Shoreit A. Study of enhancement and inhibition phenomena and genes relating to degradation of petroleum polycyclic aromatic hydrocarbons in isolated bacteria. Microbiology. 2014;83:599–607. doi: 10.1134/s002626171405012925844471

[20] Artegoitia VM, Foote AP, Lewis RM, Freetly HC. Rumen fluid metabolomics analysis associated with feed efficiency on crossbred steers. Sci Rep. 2017;7(1):2864. doi: 10.1038/s41598-017-02856-0 28588266 PMC5460109

[21] Yang Y, Dong G, Wang Z, Wang J, Zhang Z, Liu J. Rumen and plasma metabolomics profiling by UHPLC-QTOF/MS revealed metabolic alterations associated with a high-corn diet in beef steers. PLoS One. 2018;13:e0208031. doi: 10.1371/journal.pone.0208031 30485366 PMC6261619

[22] Cater CM, Lyman CM. Effect of bound gossypol in cottonseed meal on enzymic degradation. Lipids. 1970;5:765–769. doi: 10.1007/bf02531390

[23] Smirnova GV, Oktyabrsky ON. Glutathione in bacteria. Biochemistry (Mosc). 2005;70:1199–1211. doi: 10.1007/s10541-005-0248-3 16336178

[24] Pastore A, Federici G, Bertini E, Piemonte F. Analysis of glutathione: implication in redox and detoxification. Clin Chim Acta. 2003;333:19–39. doi: 10.1016/s0009-8981(03)00200-6 12809732

[25] Abou-Donia MB, Lyman CM, Dieckert JW. Metabolic fate of gossypol: the metabolism of^14^C‐gossypol in rats. Lipids. 1970;5:938–946. doi: 10.1007/bf02531126 5484207

[26] Han L-L, Shao H-H, Liu Y-C, Liu G, Xie C-Y, Cheng X-J, et al. Transcriptome profiling analysis reveals metabolic changes across various growth phases in *Bacillus pumilus* BA06. BMC Microbiol. 2017;17(1):156. doi: 10.1186/s12866-017-1066-7 28693413 PMC5504735

[27] Li T, Chang D, Xu H, Chen J, Su L, Guo Y, et al. Impact of a short-term exposure to spaceflight on the phenotype, genome, transcriptome and proteome of *Escherichia coli*. Int J Astrobiology. 2015;14:435–444. doi: 10.1017/s1473550415000038

[28] Du R-L, Chow H-Y, Chen YW, Chan P-H, Daniel RA, Wong K-Y. Gossypol acetate: A natural polyphenol derivative with antimicrobial activities against the essential cell division protein FtsZ. Front Microbiol. 2023;13. doi: 10.3389/fmicb.2022.1080308 36713210 PMC9878342

[29] Zhang Y, Shi H, Gu J, Jiao Y, Han S, Akindolie MS, et al. Anthraquinone-2,6-disulfonate enhanced biodegradation of dibutyl phthalate: Reducing membrane damage and oxidative stress in bacterial degradation. Bioresour Technol. 2020;302:122845. doi: 10.1016/j.biortech.2020.122845 32000129

[30] Zheng M, Wang X, Templeton LJ, Smulski DR, LaRossa RA, Storz G. DNA microarray-mediated transcriptional profiling of the *Escherichia coli* response to hydrogen peroxide. J Bacteriol. 2001;183:4562–4570. doi: 10.1128/jb.183.15.4562-4570.2001 11443091 PMC95351

[31] Farr SB, Touati D, Kogoma T. Effects of oxygen stress on membrane functions in *Escherichia coli*: role of HPI catalase. J Bacteriol. 1988;170:1837–1842. doi: 10.1128/jb.170.4.1837-1842.1988 2832383 PMC211039

[32] Seaver LC, Imlay JA. Alkyl hydroperoxide reductase is the primary scavenger of endogenous hydrogen peroxide in *Escherichia coli*. J Bacteriol. 2001;183:7173–7181. doi: 10.1128/jb.183.24.7173-7181.2001 11717276 PMC95566

[33] Miranda-Vizuete A, Damdimopoulos AE, Gustafsson J-Å, Spyrou G. Cloning, expression, and characterization of a novel *Escherichia coli* Thioredoxin. J Biol Chem. 1997;272:30841–30847. doi: 10.1074/jbc.272.49.30841 9388228

[34] Ritz D, Patel H, Doan B, Zheng M, Åslund F, Storz G, et al. Thioredoxin 2 is involved in the oxidative stress response in *Escherichia coli*. J Biol Chem. 2000;275:2505–2512. doi: 10.1074/jbc.275.4.2505 10644706

[35] Teixeira MC, Telo JP, Duarte NF, Sá-Correia I. The herbicide 2,4-dichlorophenoxyacetic acid induces the generation of free-radicals and associated oxidative stress responses in yeast. Biochem Biophys Res Commun. 2004;324:1101–1107. doi: 10.1016/j.bbrc.2004.09.158 15485668

[36] Chiang SM, Schellhorn HE. Regulators of oxidative stress response genes in *Escherichia coli* and their functional conservation in bacteria. Arch Biochem Biophys. 2012;525:161–169. doi: 10.1016/j.abb.2012.02.007 22381957

[37] Wong RC, Nakagawa Y, Perlmann GE. Studies on the nature of the inhibition by gossypol of the transformation of pepsinogen to pepsin. J Biol Chem. 1972;247:1625–1631. doi: 10.1016/s0021-9258(19)45601-9 4551945

[38] Montamat EE, Burgos C, Gerez de Burgos NM, Rovai LE, Blanco A, Segura EL. Inhibitory Action of Gossypol on Enzymes and Growth of *Trypanosoma cruzi*. Science. 1982;218:288–289. doi: 10.1126/science.6750791 6750791

[39] Grewal J, Tiwari R, Khare SK. Secretome analysis and bioprospecting of lignocellulolytic fungal consortium for valorization of waste cottonseed cake by hydrolase production and simultaneous gossypol degradation. Waste Biomass Valorization. 2020;11:2533–2548. doi: 10.1007/s12649-019-00620-1

[40] Santana AT, Guelfi M, Medeiros HCD, Tavares MA, Bizerra PFV, Mingatto FE. Mechanisms involved in reproductive damage caused by gossypol in rats and protective effects of vitamin E. Biol Res. 2015;48(1):43. doi: 10.1186/s40659-015-0026-7 26227499 PMC4521381

[41] Mukherjee A, Das D, Kumar Mondal S, Biswas R, Kumar Das T, Boujedaini N, et al. Tolerance of arsenate-induced stress in *Aspergillus niger*, a possible candidate for bioremediation. Ecotoxicol Environ Saf. 2010;73:172–182. doi: 10.1016/j.ecoenv.2009.09.015 19811831

[42] Fei Y-Y, Bhat JA, Gai J-Y, Zhao T-J. Global transcriptome profiling of *Enterobacter* strain NRS-1 in response to hydrogen peroxide stress treatment. Appl Biochem Biotechnol. 2020;191:1638–1652. doi: 10.1007/s12010-020-03313-x 32198600

[43] El-Mansi EMT, Nimmo HG, Holms WH. Pyruvate metabolism and the phosphorylation state of isocitrate dehydrogenase in *Escherichia coli*. Microbiology. 1986;132:797–806. doi: 10.1099/00221287-132-3-797 3525743

