# Supplementary material for: Integrative transcriptomic and metabolomic analysis to elucidate the effect of gossypol on Enterobacter sp. GD5
Source: PLoS One. 2024 Aug 6;19(8):e0306597. doi: 10.1371/journal.pone.0306597 (PMC11302909; doi:10.1371/journal.pone.0306597)
Supplement: S1 Table — (DOCX) [file pone.0306597.s001.docx]

**S 1** Gene names and primer sequences

| Gene names | Primer sequences (5'-3') | Length |
| --- | --- | --- |
| *katG*-F | CGCTGACGCCATCACCTCC | 161 bp |
| *katG*-R | GCATAATTTCCGGCGCATCCAC |  |
| *dps*-F | GATCAACAGCAAAACGCCACT | 144 bp |
| *dps*-R | GCGGTATCTTCATCTTTGGCTT |  |
| *poxB*-F | ATTAATCGGCTTCTCGTCAGG | 119 bp |
| *poxB*-R | CAATCTGAATAATTTTGGCATCGG |  |
| *lsrA*-F | ATCTCCAGCGATCTTGACGAA | 107 bp |
| *lsrA*-R | CGATCCAGACTCACGGCAT |  |
| *hemH*-F | GATTACGCCGATAACCACGA | 108 bp |
| *hemH*-R | AATGCCATGATAAGAGAGCAG |  |
| *UfaA1*-F | ATGAGGATCTAAGAACTGCACA | 174bp |
| *UfaA1*-R | ATCCTAGCTCTAAGACCCCAT |  |
| 16S-F | CCGCATAACGTCGCAAGA | 143 bp |
| 16S-R | AGTGTGGCTGGTCATCCT |  |
