# Supplementary material for: Integrative transcriptomic and metabolomic analysis to elucidate the effect of gossypol on Enterobacter sp. GD5
Source: PLoS One. 2024 Aug 6;19(8):e0306597. doi: 10.1371/journal.pone.0306597 (PMC11302909; doi:10.1371/journal.pone.0306597)
Supplement: S1 Fig — (DOCX) [file pone.0306597.s006.docx]

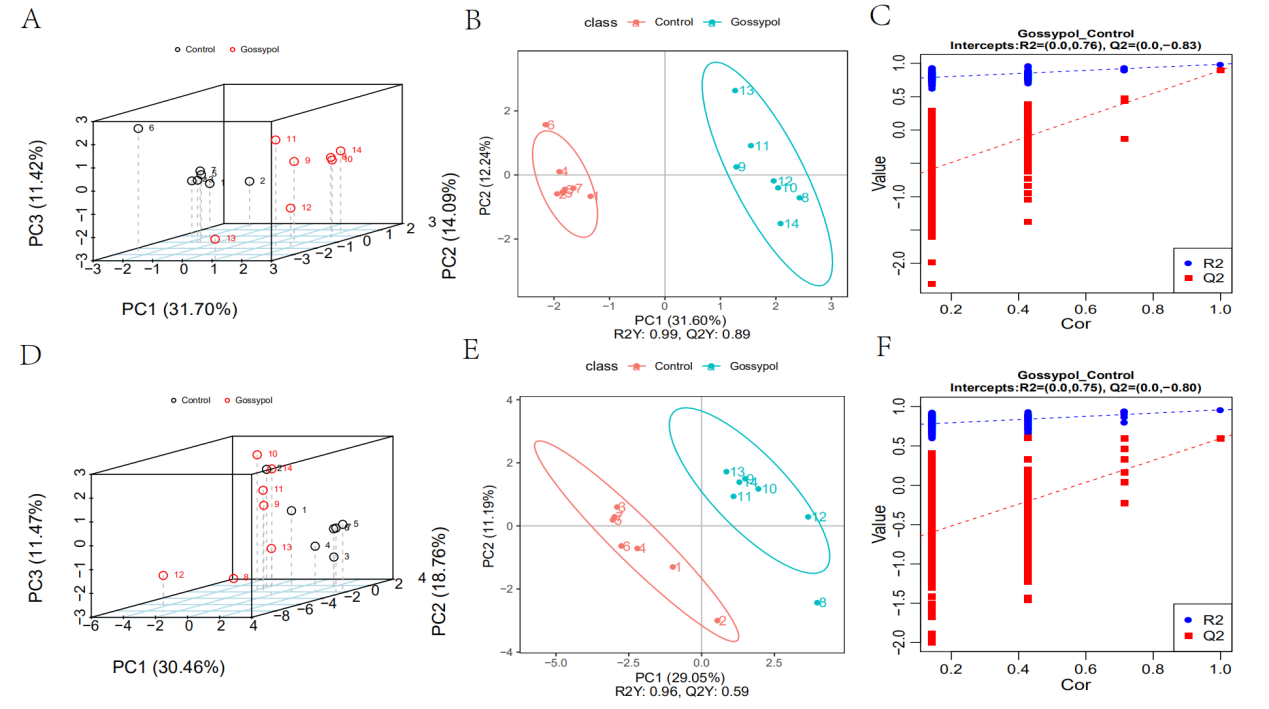


**S2 Fig.** PCA scatter plots, PLS-DA, and permutation tests between gossypol-treated and control groups. PCA scatter plots (A and D), PLS-DA results (B and E), and permutation tests (C and F). Results derived from (A–C) positive ion mode and (D–F) negative ion mode.
