# Supplementary material for: Integrative transcriptomic and metabolomic analysis to elucidate the effect of gossypol on Enterobacter sp. GD5
Source: PLoS One. 2024 Aug 6;19(8):e0306597. doi: 10.1371/journal.pone.0306597 (PMC11302909; doi:10.1371/journal.pone.0306597)
Supplement: S2 Fig — (DOCX) [file pone.0306597.s007.docx]

**S5** Test results of total RNA

| Samples | Concentration(ng/μL) | Total concentration(μg) | OD_260/280_ | OD_260/230_ | RIN |
| --- | --- | --- | --- | --- | --- |
| Con-1 | 92 | 3.036 | 1.58 | 1.50 | 7.9 |
| Con-3 | 256 | 8.192 | 1.94 | 2.33 | 8.6 |
| Con-4 | 144 | 4.608 | 2.00 | 1.50 | 9.5 |
| Con-5 | 164 | 5.248 | 2.05 | 0.81 | 9.5 |
| Con-6 | 110 | 3.52 | 1.83 | 1.45 | 9.5 |
| Con-7 | 224 | 7.168 | 2.00 | 1.53 | 9.9 |
| Gos-1 | 706 | 22.592 | 2.05 | 2.35 | 9.4 |
| Gos-3 | 1,088 | 34.816 | 2.09 | 2.20 | 9.4 |
| Gos-4 | 386 | 12.352 | 2.10 | 2.33 | 9.2 |
| Gos-5 | 1,266 | 40.512 | 2.09 | 2.21 | 10.0 |
| Gos-6 | 834 | 26.688 | 2.08 | 2.24 | 10.0 |
| Gos-7 | 1,248 | 39.936 | 2.09 | 2.15 | 6.0 |

Note: RIN (RNA Integrity Number) is frequently used evaluate of RNA quality. The score is from 0 to 10. Higher score means better RNA integrity.
