# Supplementary figures and images for: Integrative transcriptomic and metabolomic analysis to elucidate the effect of gossypol on Enterobacter sp. GD5
Source: PLoS One. 2024 Aug 6;19(8):e0306597. doi: 10.1371/journal.pone.0306597 (PMC11302909; doi:10.1371/journal.pone.0306597)

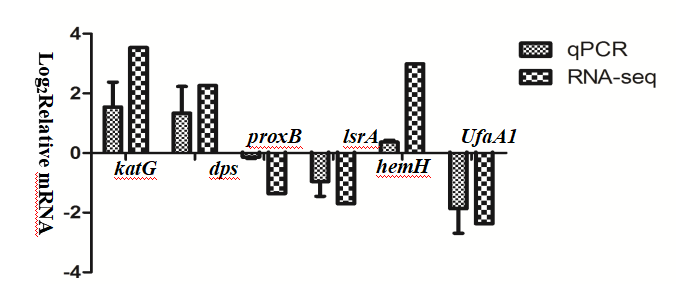
S8

Supplement: S3 Fig — (DOC) [file pone.0306597.s008.doc]
